# Reproductive and Environmental Drivers of Time and Activity Budgets of Striped Skunks

**DOI:** 10.1093/iob/obz013

**Published:** 2019-06-14

**Authors:** V Y Zhang, C T Williams, T C Theimer, C Loren Buck

**Affiliations:** 1 Department of Biological Sciences, Northern Arizona University, Flagstaff, AZ 86011, USA; 2 Institute of Arctic Biology and Department of Biology & Wildlife, University of Alaska Fairbanks, Fairbanks, AK 99775, USA

## Abstract

The regulation of daily and circannual activity patterns is an important mechanism by which animals may balance energetic requirements associated with both abiotic and biotic variables. Using collar-mounted accelerometers, we assess the relative importance of reproductive stage and environmental conditions on the overall dynamic body acceleration (ODBA) of free-living striped skunks (*Mephitis mephitis*). We found that activity timing relative to photoperiod varied across seasonal stages for both sexes. Surprisingly, male skunks did not commence activity earlier than females during the mating interval. Moreover, while female skunks began activity before dusk and terminated activity after dawn during mid- through late summer (lactation period), the duration of activity bouts in females during this period was not different from other seasons. Both male and female skunks exhibited high variability and fragmentation in daily activity rhythms except during the lactation period, when females appear to switch to prolonged bouts of nocturnal activity. Overall, ODBA varied by season and sex, with changes in ODBA indicative of seasonal reproductive requirements such as conspecific competition for mates in males and lactation in females. Weather conditions had little effect on skunk activity levels except during the winter season, when snow cover and temperature negatively influenced daily ODBA. Taken together, the activity patterns of striped skunks appear to be primarily driven by seasonal investment in reproduction and secondarily by thermoregulatory constraints during the non-winter months. Our results highlight the importance of considering how environmental and reproductive drivers may interact to affect activity across both the daily and seasonal cycle.

## Synopsis

Regulación de la energía en mofetas rayadas ante la reproduccin y el ambiente La regulación de los patrones de actividad diaria y circanual es un mecanismo importante mediante el cual los animales pueden equilibrar los requerimientos energéticos asociados con las variables bióticas y abióticas. Usando acelerómetros montados en collar, evaluamos la importancia relativa de la etapa reproductiva y las condiciones ambientales en la aceleración global dinámica del cuerpo (ODBA, por sus siglas en inglés) de las mofetas rayadas (Mephitis mephitis). Encontramos que el tiempo de actividad en relación con el fotoperíodo varió a lo largo de las etapas estacionales para ambos sexos. Sorprendentemente, las mofetas macho no comenzaron la actividad antes que las hembras durante el intervalo de apareamiento. Además, las mofetas hembra comenzaron la actividad antes del anochecer y terminaron la actividad después del amanecer durante la mitad del verano (período de lactancia), la duración de los episodios de actividad en las hembras durante este período no fue diferente de otras estaciones. Tanto las mofetas macho como las hembra mostraron una alta variabilidad y fragmentación en los ritmos de la actividad diaria, excepto durante el período de lactancia, cuando las hembras parecen cambiar a episodios prolongados de actividad nocturna. En general, la ODBA varió según la temporada y el sexo, con cambios en la ODBA indicativos de los requisitos reproductivos estacionales, como la competencia específica para los machos en los machos y la lactancia en las hembras. Las condiciones climáticas tuvieron poco efecto en los niveles de actividad de la mofeta, excepto durante la temporada de invierno, cuando la capa de nieve y la temperatura influyeron negativamente en la ODBA diaria. Tomados en conjunto, los patrones de actividad de las mofetas rayadas parecen estar impulsados principalmente por la inversión estacional en la reproducción y, en segundo lugar, por las restricciones de termorregulación durante los meses que no son de invierno. Nuestros resultados resaltan la importancia de considerar cómo los impulsores ambientales y reproductivos pueden interactuar para afectar la actividad a lo largo del ciclo diario y estacional. Translated to Spanish by S Hinojosa (hinojosa.silvia@gmail.com)

Fortpflanzungs- und Umwelttriebkräfte für Zeit- und Aktivitätsbudgets von StreifenskunksDie Regulierung der Aktivitätsmuster im Tages- und Jahresrythmus ist ein wichtiger Mechanismus, durch den Tiere den energetischen Bedarf ausgleichen können, der sowohl im Zusammenhang mit abiotischen als auch biotischen Variablen entsteht. Mithilfe von an Halsbändern befestigten Beschleunigungsmessern beurteilen wir die relative Bedeutung des Reproduktionsstadiums und der Umweltbedingungen für die dynamische Gesamtkörperbeschleunigung (overall dynamic body acceleration, ODBA) von frei lebenden Streifenskunks (Mephitis mephitis). Wir fanden heraus, dass die zeitliche Abstimmung der Aktivität in Bezug auf die Photoperiode für beide Geschlechter saisonal variierte. Überraschenderweise waren männliche Stinktiere während des Paarungsintervalls nicht früher aktiv als weibliche. Während weibliche Stinktiere außerdem Mitte des Sommers bis in den Spätsommer (Laktationsperiode) vor der Dämmerung aktiv waren und ihre Aktivität nach Sonnenaufgang beendeten, unterschied sich die Dauer der Aktivitätsphasen der Weibchen während dieser Periode nicht von anderen Jahreszeiten. Sowohl männliche als auch weibliche Stinktiere zeigten eine hohe Variabilität und Fragmentierung im täglichen Aktivitätsrhythmus, außer während der Laktationsperiode, wenn die Weibchen zu längeren nächtlichen Aktivitätsphasen zu wechseln scheinen. Insgesamt variierte die ODBA je nach Saison und Geschlecht. Die Veränderungen der ODBA deuten auf saisonale Fortpflanzungserfordernisse hin, wie etwa die konspezifische Konkurrenz um Partner bei männlichen Tieren und die Laktation bei weiblichen Tieren. Die Wetterbedingungen hatten nur einen geringen Einfluss auf die Aktivität der Stinktiere, außer in der Wintersaison, wo die Schneedecke und die Temperatur die tägliche ODBA negativ beeinflussten. Zusammengefasst scheinen die Aktivitätsmuster von Streifenskunks in erster Linie durch saisonale Investitionen in die Fortpflanzung und sekundär durch thermoregulatorische Einschränkungen in den Nicht-Wintermonaten bedingt zu sein. Unsere Ergebnisse zeigen, wie wichtig es ist in Betracht zu ziehen, wie Umwelt- und Fortpflanzungstriebkräfte interagieren könnten um die Aktivität im täglichen und saisonalen Zyklus zu beeinflussen. Translated to German by F Klimm (frederike.klimm@biologie.uni-freiburg.de)

## Introduction

Predictable energetic costs incurred across an animal’s life can be broadly divided into those imposed by abiotic changes in their environment (e.g., temperature, photoperiod, and precipitation) (Forrest and Miller-Rushing 2010) and biotic changes in physiology and behavior. Animals that inhabit highly seasonal environments, such as those at high latitudes and altitudes, typically experience greater seasonal fluctuations than their lower latitude and altitude counterparts and thus experience significant constraints on time–energy budgets across the year. In addition, many species undergo predictable seasonal changes in physiology and behavior associated with reproduction. These physiological changes are accompanied by significant differences in an animal’s energetic requirements with the timing of peak energy demand often differing between sexes. As the relative importance of thermoregulatory and reproductive demands fluctuate across the day and year, how animals concomitantly allocate their time and energy determines their survival and reproductive success. Given that movement-based energy expenditure contributes significantly to total energy expenditure (Brown et al. 2013), many have hypothesized that animals behaviorally compensate via regulation of activity patterns (Gittleman and Thompson 1988; Logan and Sanson 2003; Terrien et al. 2011). Examining how animals regulate activity on both daily and seasonal time scales is necessary to understand the energetic consequences of adjustments to activity budgets.

The daily light:dark cycle is one of the most predictable synchronizing agents of daily activity patterns of animals. The timing of activity across the day influences the fitness costs and benefits of many behaviors (Daan and Aschoff 1982). Some small mammals adjust their timing of activity seasonally to consolidate activity during the time of day that is thermally most efficient (Tachinardi et al. 2017; Kenagy 2002) or to match resource availability (Hut et al. 2011; van der Vinne et al. 2015). Within a species, individuals can vary in their propensity to be active at particular times of the day, referred to as their “chronotype” (Horne and Östberg 1976; Roenneberg et al. 2007; Hau et al. 2017). Moreover, the timing of daily activity has been argued to be a sexually selected character trait in mammals, and sexual selection theory predicts that males should initiate daily activity earlier during the mating season relative to the rest of the year, as they seek to intercept receptive females (Hau et al. 2017). Timing as a sexually selected trait has been studied primarily in birds, with the most prominent example being the dawn song of male birds. Variation in the timing (substrate of sexual selection) of the onset of dawn singing is correlated with variation in extra-pair paternity (Krebs and Kacelnik 1983; Poesel et al. 2006; Kempenaers et al. 2010). Currently, there is a dearth of empirical evidence on whether chronotype is a sexually selected trait in mammals due to the difficulties associated with gathering fine-resolution data on daily patterns of individual activity.

Seasonal life cycle transitions are associated with changes in physiology and behavior that allow animals to meet environmental and reproductive demands. For example, in temperate environments, the winter activity levels of mammals are often lower than in other seasons due to factors including snow cover (Raine 1983) and unfavorable thermoregulatory conditions (O’Farrell 1974; Owen-Smith 1998). Also, when negative energy balance can be anticipated because of predictable seasonal environmental changes, some species use daily torpor or seasonal hibernation to conserve energy (Ruf and Geiser 2015). However, the relative importance of thermoregulatory versus reproductive requirements will shift with onset of the breeding season, and sex-specific differences in the timing of energy allocation toward reproduction often results in time-activity budgets that differ between sexes. Breeders will begin to allocate more time and energy toward reproductive effort as food availability increases and thermoregulatory costs decrease (Massei et al. 1996; Geisser and Reyer 2005; Persson 2005). In species with polygynous and promiscuous mating systems, the most important resources for males are hypothesized to be receptive females during the breeding season and food during the non-breeding season. In contrast, successful reproduction by females is linked to their ability to acquire food during critical parts of the year to meet elevated maternal and offspring energy requirements (Erlinge and Sandell 1986). Yet, how sex-specific differences in reproductive effort relate to the intensity or timing of activity in polygynous/promiscuous species has rarely been investigated quantitatively.

One logistical challenge to understanding activity patterns in free-living mammals is that tools such as camera-trapping, live-trapping, and radio tracking, do not provide continuous information on an animal’s activity across the day and year. Generally, studies on the biological timing of activity require long term and precise data to adequately estimate daily and seasonal trends (Hau et al. 2017). The reduction in size and cost of biologging devices, such as accelerometers, now enable researchers to understand the fine-scale activity patterns of mammals (Williams et al. 2016a). Accelerometers continuously measure and record acceleration on three orthogonal axes and provide an integrated measure of overall dynamic body acceleration (ODBA) (Halsey et al. 2008, 2009). ODBA reflects total movement of an animal and increases with increasing activity levels. The strong correlation between ODBA and activity-specific energy expenditure has been demonstrated in a range of mammals (Halsey et al. 2009; Pagano and Williams 2019), birds (Wilson et al. 2006), amphibians (Halsey and White 2010), and reptiles (Halsey et al. 2011).

In North America, the striped skunk (*Mephitis mephitis*) is a common and abundant nocturnal mesocarnivore that likely experiences significant sex-specific differences in energetic requirements across the year (Storm 1972; Sunquist 1974; Larivière and Messier 1997; Weissinger et al. 2009; Neiswenter et al. 2010). As polygynous/promiscuous breeders, male skunks actively search for mating opportunities with multiple females during the mating season. In captivity, females show aggressive behavior toward males soon after mating (Verts 1967), so mate guarding, if it occurs, is likely to be brief and males should maximize the number of receptive females they can encounter. Mating activities of male skunks typically begin in early spring and last 4 weeks (Ernst 1965). Gestation is ∼68 days, with parturition occurring during late spring–early summer (Wade-Smith and Verts 1982). Females are the sole contributors to the care of young (Wade-Smith and Verts 1982) and the altricial young are nursed in the maternal den for 6 weeks (Larivière and Messier 1997). Young are at heel by mid- to late-summer, then disperse and begin fattening for the subsequent winter by fall (Wade-Smith and Verts 1982; Larivière and Messier 1997).

Given the solitary and secretive nature of many nocturnal carnivores, it is difficult to robustly sample their activity patterns. Here, we deployed accelerometers on striped skunks to understand how reproductive and environmental drivers may interact to influence patterns of activity across the day and year. In related hog-nosed skunks (*Conepatus chinga*), ODBA has been shown to be a proxy for oxygen consumption (Halsey et al. 2009). Because timing of daily activity is likely a sexually selected trait in mammals (Hau et al. 2017), we hypothesized that males would initiate activity earlier in the day during the mating season than during other times of year. We further hypothesized that activity levels of striped skunks would reflect sex-specific differences in timing and energy allocation toward reproduction. To better infer the physiological significance of activity and its association to overall energy balance in skunks, we also examined the timing of activity onset and offset, duration of activity bouts, and distribution of activity patterns (activity timing) across the day. Reproductive females have been shown to increase their foraging efforts during the energetically costly period of lactation (Kenagy et al. 1989; Fletcher et al. 2012). Thus, we predicted that daily activity of female skunks during lactation would be higher than during other seasons. As males compete for access to multiple females, we also predicted that activity of reproductive males would be greatest during the spring mating interval. Lastly, we hypothesized that unfavorable weather such as cold and wet conditions would decrease activity of both male and female skunks.

## Methods

### Study area

This study was conducted in a low- to medium-density suburban neighborhood of Flagstaff, Arizona (35°11′57′′N 111°37 ′52′′W). At 2170 m elevation, Flagstaff is nested within the largest contiguous ponderosa pine forest in the world (Cooper 1960). Flagstaff experiences a seasonal climate that includes subfreezing temperatures and heavy snowfall during winter and a wet monsoonal climate during summer, when the majority of the city’s average precipitation (55 cm) in the form of rainfall occurs (Staudenmaier et al. 2014). The study area was ∼5.5 km^2^ and included residential areas mixed with wooded natural areas and several golf courses. The southern and eastern borders of the neighborhood bordered extensive forested wildlands.

### Collar deployments

We studied a free-living population of suburban striped skunks (*M.mephitis* hereafter skunks) between October 30, 2015 and October 30, 2016. We performed four separate device deployments on skunks, representative of four different seasonal stages of activity: Winter November 13–December 30 (four males, four females), Mating February 29–March 22 (four males, three females), Lactation/Young at Heel June 26–July 26 (three females), and Fattening/Dispersal August 18–September 18 (two males, four females). No males were captured from June through July (Lactation/Young at Heel stage).

Skunks were captured using live-traps (Tomahawk Live Trap, Models 106 and 108, Tomahawk, WI) baited with peanut butter or canned tuna. Traps were set at dusk and checked the following morning. When trapping success was low, we used hand-held nets to capture animals as they left their dens at night. Upon capture, skunks were transported to Northern Arizona University (NAU) for processing and held in a shed with *ad libitum* food and water. Animals were released at the site of capture the following evening.

Skunks were anesthetized using a 0.5 mL intramuscular injection of 5:1 ketamine/xylazine and assessed for sex and reproductive status; animals were also sampled for blood (∼0.5 mL) via toenail clip (Lee et al. 2011) for use in other studies. We deployed radio-collars affixed with accelerometers wrapped in heat-shrink tubing (axy-3 loggers, TechnoSmart Europe srl., Rome, Italy) on 21 individuals (11 females, 10 males) across four deployment periods, resulting in a total of 772 animal-days of usable acceleration data. During the first deployment, exactly 48 days of data were collected from each animal, during the second deployment 19 ± 6.3 (standard deviation [SD]) days, during the third deployment exactly 31 days, and during the fourth deployment 28 ± 5.6 days. Animals from the first deployment were used in a separate study (Theimer et al. 2017b); these individuals were outfitted with proximity-sensing loggers and implanted with body temperature loggers in addition to accelerometers. Total collar weight was ∼40g (<2% body mass; includes weight of proximity-sensing logger, radio transmitter, and accelerometer) during the first deployment and 11 g (<1% body mass; includes weight of radio transmitter and accelerometer) during the second through fourth deployments. Average animal weight was 2.6 ± 0.7 (SD) kg. All captured animals were uniquely marked with numbered aluminum ear tags for identification (#1005-1 National Band and Tag Company, Newport, Kentucky). Efforts to recapture animals and remove collars began 2 months after initial deployment during the first winter deployment and 10–14 days after initial deployment for the second through fourth deployments. This study followed the guidelines of American Society of Mammologists (Sikes et al. 2016).

### Accelerometers

Accelerometers were programmed to measure and record acceleration on three orthogonal axes (X, Y, Z) at either 1 hz or 10 hz; all accelerometers from deployment 1, one from deployment 2, and five devices from deployment 4 recorded at 1 hz. We subtracted the running mean (5 s for 1 hz, 3 s for 10 hz) of acceleration on each axis to compensate for the static effect of gravity. ODBA was calculated by summing the gravity-adjusted absolute values of acceleration on all three axes. To ensure that 1 hz sampling rates provide a similar measure of ODBA as 10 hz, we used simple linear regression of hourly averages of activity at the two sampling frequencies and found no significant differences (Intercept = 6.72 × 10^−4^, Estimate = 0.988, *R*^2^ = 0.998) (Supplementary Fig. S1).

### Environmental data

We used the weatherData R package (Narasimhan 2017) to interrogate weather data from the Weather Underground database (Weather Station ID: KAZFLAGS40, Hardware: Davis Vantage Pro2). The weather station was programmed to measure and record data every 5 min and we examined environmental parameters known to affect thermal exchange rates and movement in small mammals including: ambient temperature, precipitation, snow cover, wind speed, and the interaction between wind speed and ambient temperature (i.e., wind chill effect) (Williams et al. 2014; 2016b). Snow cover data was obtained from Flagstaff Pulliam Airport through the National Oceanic and Atmospheric Administration online database (https://www.ncdc.noaa.gov/cdo-web/). Information on photoperiod was obtained from the Astronomical Applications Department of the US Naval Observatory.

### Statistical analysis

All analyses were performed with R statistical software (version 3.2.4; http://www.R-project.org/). We used linear mixed-effects models with mean daily ODBA as our response variable and individual animal and day of year as random effects to examine the activity patterns of skunks across the year (package lmerTest; Kuznetsova et al. 2017). For all models, we assessed normality of the residuals using quantile–quantile plots. Due to significant covariance between seasonal stage and environmental conditions (*P* < 0.01 for all weather parameters), these effects could not co-occur in the same mixed model. Thus, we first ran two models that included data from across the entire year (determining stage effects for each sex) and four other models that examined each of the four seasonal stages separately (determining weather and sex effects within each stage). Because our design was unbalanced with no measures from males during the third deployment, the first two mixed models separately examined the influence of seasonal stage on mean daily ODBA for each sex with breeding stage as a main effect. To examine effects of environmental parameters and sex on mean daily ODBA, we used one mixed model for each of the four recorded seasonal stages, including sex and weather parameters as main effects. Snow cover was included as a categorical variable (snow cover ≥8 cm or snow cover <8 cm). Due to the highly-skewed distribution of rainfall volumes, we used the square root of precipitation rate in our analysis. Snow-cover was only present during the winter period and rainfall was only present during the late lactation and fattening/dispersal period. Daily averages of all other weather conditions (ambient temperature, rainfall, wind speed and wind chill) were fixed effects in our models of ODBA.

The “activity on- and offset” tool from ActogramJ was used to determine the initiation and termination times of dawn and dusk-related locomotion (activity onset/offset) using Gaussian kernel smoothing (smoothing Gaussian SD = 5 min; threshold = median) (http://actogramj.neurofly.de/; Schmid et al. 2011) and to generate actograms for each skunk. To investigate changes in activity onset and offset relative to photoperiod, we used civil twilight (dusk and dawn) as a reference frame. The response variable (activity onset/offset time relative to civil twilight) was calculated by subtracting the time of activity onset/offset from the time of civil twilight for each animal. Due to the high variability and internal fragmentation in day-to-day activity rhythms of animals across the year (Supplementary Fig. S2), ActogramJ was unable to accurately detect onset/offset times on days when activity was low or ultradian. In order to remove spurious detections, we limited detections to times likely relevant to skunk activity cycles and only included activity onsets that occurred between 15:00 and 23:00 and activity offsets between 1:00 and 9:00 in our analysis. Duration of daily skunk activity bouts was calculated as the difference between time of activity offset and activity onset. During the winter period, only 17.4% of days had detectable onset/offset times when snow-cover was 8 cm or above; therefore, we did not include days with snow-cover when analyzing activity timing. We used multiple linear mixed-effects models to determine the effect of seasonal stage separate from weather and sex effects as previously described. Weather was averaged over a 5-h window centered on the time of civil twilight for each day; this 5-h window coincides with >95% of activity onsets. All significant differences between computed least-square means across breeding stages were determined using pairwise Tukey post-hoc tests. All differences were considered significant when *P* ≤ 0.05.

### Ethics statement

This study adhered to animal-care protocols and was approved by the NAU institutional animal Use and Care Committee (Protocol 11-002-R1).

## Results

### Activity timing

**Fig. 1:**
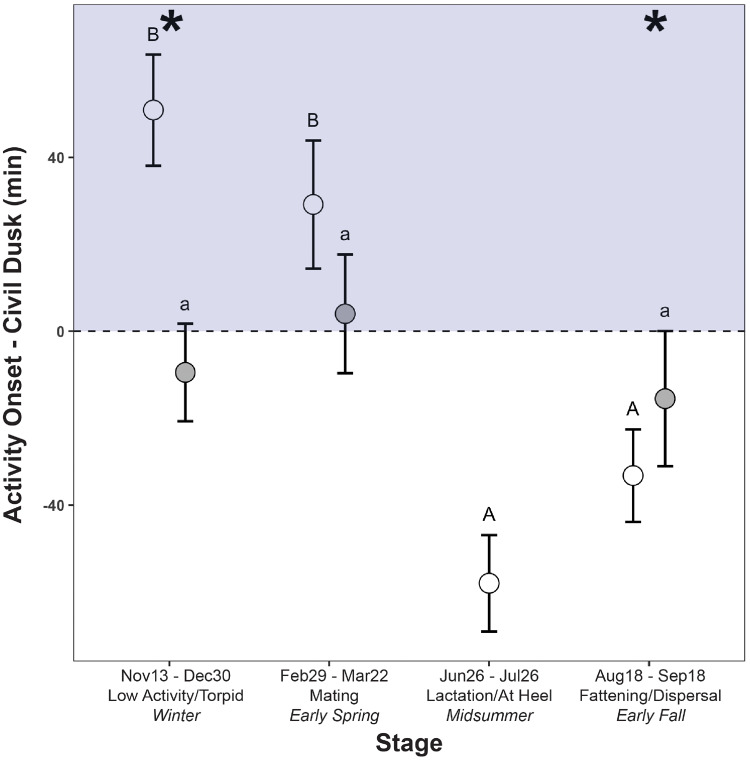
Time of daily activity onset (LS means ± SE) in female (open circles) and male (closed circles) striped skunks relative to the timing of civil dusk during four seasonal stages across the annual cycle. Dashed line represents civil dusk time. Positive values (gray fill/nighttime) represent activity onset beginning after civil dusk and negative values (white/daytime) before. Asterisks represent significant differences in activity onset between sexes. Uppercase letters represent significant differences in timing of activity onset for females and lowercase for males, as determined by *post hoc* tests (*P* < 0.05).

Onset times of daily activity bouts relative to timing of civil dusk varied by seasonal stage in females (F_3,29_ = 18.93, *P* < 0.0001) but not males (F_2,11_ = 0.50, *P* = 0.62) ((b) in Table 1). Timing of activity onset in females was significantly later during the winter and the mating period as compared to lactation/at heel and during fattening (Fig. 1). The onset of female activity bouts occurred after civil dusk during the winter (least squares means = 50.9 ± 12.8 [standard error; SE] min after civil dusk) and during the mating period (29.2 ± 14.7 min). During lactation/at heel and fattening, female activity onsets occurred earlier in the day, before civil dusk (lactation: −58.0 ± 11.1 min; fattening: −33.2 ± 10.7 min). Activity onsets for males were slightly earlier than civil dusk during the fattening stage (−15.5 ± 15.6 min) and were significantly earlier than females during the winter period (F_1,8_ = 7.65, *P* = 0.03) and during the fattening period (F_1,148_ = 7.61, *P* = 0.01).

**Table 1:** Parameter estimates (95% CI and *P*-values) for the influence of seasonal stage on ODBA (a), activity onset (b), activity offset (c), and activity period (d) in striped skunks, as determined by mixed models.

Sex		Parameter	Estimate (95% CI)	*P*-value	Random effects	
(a)	Female	Stage				
		Winter	0		σ^2^	0.001
		Mating	0.028 (0.014 to 0.04)	<0.0001	τ_00_ _Day of Year_	0.0001
		Lactation	0.15 (0.14 to 0.16)	<0.0001	τ_00_ _Individual_	0.001
		Fattening	0.04 (0.03 to 0.05)	<0.0001		
	Male	Stage				
		Winter	0		σ^2^	0.001
		Mating	0.09 (0.066 to 0.113)	<0.0001	τ_00_ _Day of Year_	0.001
		Fattening	−0.01 (−0.035 to 0.016)	0.475	τ_00_ _Individual_	0.002
(b)	Female	Stage				
		Winter	0		σ^2^	2855
		Mating	−21.8 (−59.8 to 16.3)	0.27	τ_00_ _Day of Year_	2010
		Lactation	−108.9 (−140.3 to −77.6)	<0.0001	τ_00_ _Individual_	115
		Fattening	−84.15 (−115.2 to −53.1)	<0.0001		
	Male	Stage				
		Winter	0		σ^2^	4213
		Mating	13.5 (−21.2 to 48.2)	0.46	τ_00_ _Individual_	302
		Fattening	−6.0 (−43.7 to 31.6)	0.76		
(c)	Female	Stage				
		Winter	0		σ^2^	3085
		Mating	59.6 (19.5 to 99.7)	0.01	τ_00_ _Individual_	769
		Lactation	165 (140 to 190)	<0.0001		
		Fattening	106 (80.7 to 131 )	<0.0001		
	Male	Stage				
		Winter	0		σ^2^	4537
		Mating	134 (67.1 to 201 )	0.002	τ_00_ _Day of Year_	248
		Fattening	83.2 (5.23 to 161 )	0.06	τ_00_ _Individual_	1880
		Stage				
(d)	Female	Winter	0		σ^2^	1.383
		Mating	−0.69 (−1.21 to −0.17)	0.01	τ_00_ _Day of Year_	0.007
		Lactation	−0.22 (−0.65 to 0.21)	0.31		
		Fattening	0.22 (−0.21 to 0.64)	0.32		
		Stage				
	Male	Winter	0		σ^2^	2.08
		Mating	0.32 (−0.63 to 1.28)	0.52	τ_00_ _Individual_	0.306
		Fattening	−1.30 (−2.37 to −0.22)	0.04		

CI, confidence interval.

**Fig. 2:**
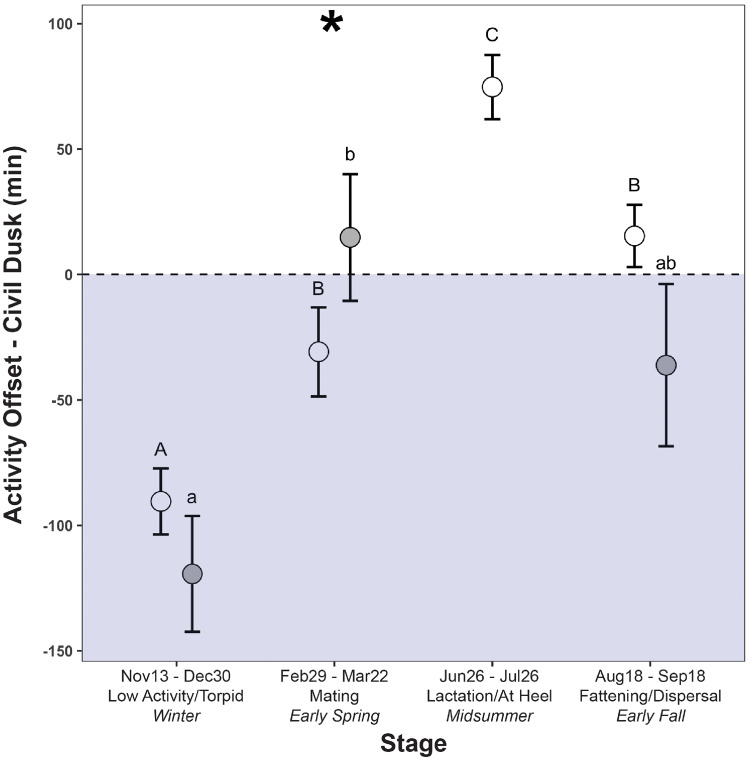
Time of daily activity offset (LS means ± SE) in female (open circles) and male (closed circles) striped skunks relative to the timing of civil dawn during four separate seasonal stages across the annual cycle. Dashed line represents civil dusk time. Positive values (white/daytime) represent activity offset beginning after civil dawn and negative values (gray fill/nighttime) before. Asterisks represent significant differences in activity offset between sexes. Uppercase letters represent significant differences in timing of activity offset for females and lowercase for males, as determined by *post hoc* tests (*P* < 0.05).

Both males (F_2,11_ = 7.86, *P* = 0.01) and females (F_3,37_ = 55.78, *P* < 0.0001) varied their activity cessation times relative to timing of civil dawn by seasonal stage ((c) in Table 1). Both sexes appear to terminate activity before dawn during the winter period and around the time of dawn during mating and fattening, with males terminating activity significantly later than females during the mating period (F_1,85_ = 8.38, *P *= 0.01) (Fig. 2). During lactation, females terminated activity 74.7 ± 12.8 min after civil dawn.

**Fig. 3:**
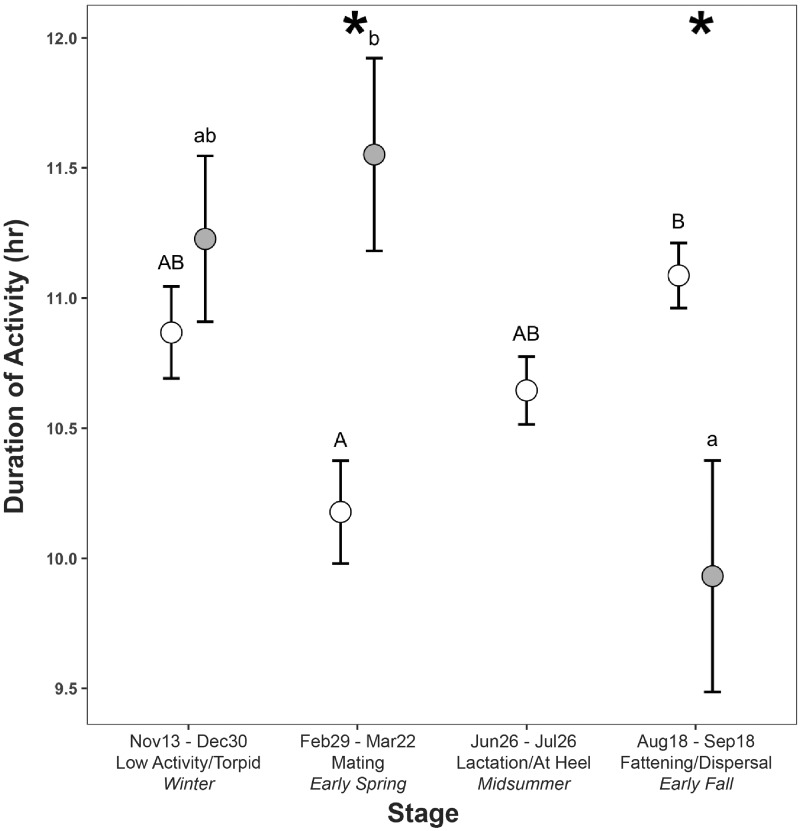
Duration of daily activity bouts (LS means ± SE) in female (open circles) and male (closed circles) striped skunks during four separate seasonal stages across the annual cycle. Asterisks represent significant differences in activity duration between sexes. Uppercase letters represent significant differences in activity duration for females and lowercase for males, as determined by *post hoc* tests (*P* < 0.05).

Duration of daily activity bouts varied by season in both males (F_2,11_ = 4.23, *P* = 0.05) and females (F_3,75_ = 5.53, *P* = 0.002) ((d) in Table 1). Males had the longest activity bouts during mating and the shortest during fattening (mating: 11.6 ± 0.4 h; fattening: 9.9 ± 0.5 h); females had the longest activity bouts during fattening and shortest during mating, with bout lengths during winter and lactation being intermediate (mating: 10.18 ± 0.2 h; lactation: 10.7 ± 0.1 h; fattening: 11.1 ± 0.1 h) (Fig. 3). Males had significantly longer activity bouts than females during the mating season (F_3,37_ = 75.4, *P* = 0.002), and females were active for longer than males during fattening (F_3,37_ = 75.4, *P* = 0.002).

### Average daily activity

**Fig. 4:**
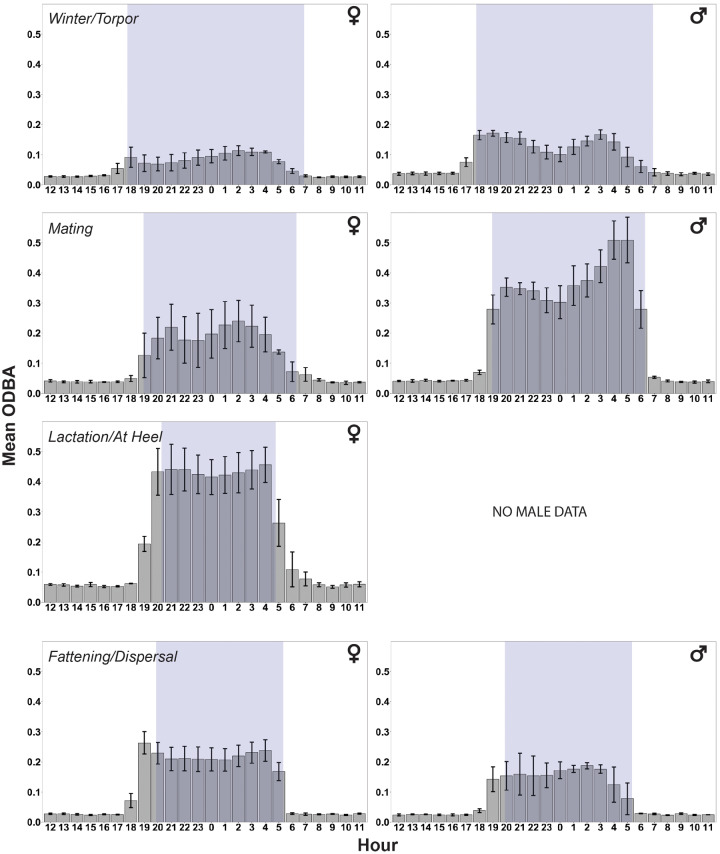
Mean (±SE) hourly ODBA of female (left panels) and male (right panels) skunks during four seasonal stages across the annual cycle. Gray shading indicates the period between civil dusk and civil dawn (nighttime) for each season.

Average daily ODBA varied by seasonal stage in both males and females (male, F_2,16_ = 35.4, *P* < 0.0001; female, F_3,358_ = 230.4, *P* < 0.0001) ((a) in Table 1). Daily ODBA was highest in females during lactation/at heel (late-June through late-July) and lowest during the winter period (mid-November through late-December [Fig. 4 and Supplementary Fig. S3]). Female ODBA during the mating interval in March was intermediate, with activity levels higher than during winter and lower than during lactation/at heel; female ODBA during lactation/at heel did not significantly differ from the activity levels during early fall (Supplementary Fig. S3). In males, daily ODBA was highest during the mating interval and lowest during the winter period and early fall (Fig. 4 and Supplementary Fig. S3). Average daily ODBA was higher in males than females during both the winter and mating periods (winter, F_1,8_ = 6.12, *P* = 0.04; mating, F_1,7_ = 9.74, *P* = 0.02). Visual examination of actograms showed that male skunks fluctuated greatly in their daily activity rhythms across the year, switching among unimodal, bimodal, and highly fragmented activity patterns. Female skunks showed a similar pattern of high variability in daily activity rhythms, except during the lactation/at heel periods and early fall, when individuals displayed a single prolonged peak in activity throughout the night (Supplementary Fig. S2). Despite the high day-to-day variability in activity rhythms observed in both males and females (Supplementary Fig. S2), females appeared on average to primarily exhibit a unimodal distribution in daily activity irrespective of season, whereas distributions in daily activity of males trended toward bimodality during winter and mating and unimodality during fattening (Fig. 4).

### Effects of weather

Despite relatively low temperatures during early-spring and increased rainfall during mid-summer, environmental conditions did not influence average daily ODBA in skunks except during the winter period (Table 2). During this time, average daily ODBA increased as ambient temperatures increased (F_1,47_ = 8.38, *P* = 0.006) and decreased significantly when snow cover was present (F_1,47_ = 20.41, *P* < 0.0001) ((a) in Table 2). During lactation, rainfall delayed the timing of activity onset (F_1,90_ = 7.71, *P* = 0.01) (Supplementary Table S1) while increased temperature and wind decreased the duration of activity bouts (temperature: F_1,90_ = 9.22, *P* = 0.003; wind: F_1,90_ = 7.86, *P* = 0.01) (Supplementary Table S2). During fattening, cessation of activity was later when ambient temperature and wind speed were higher (temperature: F_1,142_ = 21.0, *P* < 0.0001; wind: F_1,142_ = 5.0, *P* = 0.03), and activity cessation was earlier when there was rainfall (F_1,142_ = 4.5, P = 0.04) (Supplementary Table S3). We did not statistically examine the effects of snow cover on the timing or duration of activity bouts, because onset was only detectable for 17.4% of days with snow cover. Nevertheless, examination of individual actograms suggested that snow cover delayed onset times in some animals (Supplementary Fig. S2).

**Table 2:** Parameter estimates (95% CI and *P*-values) for the influence of sex and various environmental variables on ODBA in striped skunks, as determined by mixed models. Results are grouped (a–d) by four seasonal stages in which collar deployments took place

Stage		Parameter		Estimate (95% CI)	*P*-value	Random effects	
(a)	Winter/Torpor(November 13–December 30)	Sex				
		Male		0.030 (0.006 to 0.053)	0.039	σ^2^	0.001
		Female		0		τ_00_ _Day of Year_	0.00004
		Temperature (°C)		0.003 (0.001 to 0.004)	0.006	τ_00_ _Individual_	0.0003
		Wind speed (m/s)		−0.001 (−0.002 to 0.0)	0.35		
		Snow cover (cm)					
		≥8		−0.02 (−0.03 to −0.01)	<0.0001		
		<8		0			
		Temp × Wind		0.0 (−0.0003 to 0.0003)	0.97		
(b)	Mating(February 29–March 22)	Sex					
		Male		0.077 (0.029 to 0.126)	0.016	σ^2^	0.002
		Female		0		τ_00_ _Day of Year_	0.0003
		Temperature (°C)		0.0 (−0.01 to 0.01)	0.98	τ_00_ _Individual_	0.001
		Wind Speed (m/s)		−0.005 (−0.019 to 0.009)	0.5		
		Rainfall (mm^0.5^)		0.015 (−0.116 to 0.146)	0.83		
		Temp × Wind		0.001 (0.0 to 0.002)	0.52		
(c)	Lactation/at heel; Females only(June 26–July 26)	Temperature (°C)		−0.002 (−0.007 to 0.004)	0.56	σ^2^	0.0003
		Wind Speed (m/s)		0.025 (−0.010 to 0.060)	0.17	τ_00_ _Day of Year_	0.00001
		Rainfall (mm^0.5^)		0.018 (−0.016 to 0.052)	0.32	τ_00_ _Individual_	0.002
		Temp × Wind		−0.001 (−0.003 to 0.001)	0.27		
(d)	Fattening/Dispersal(August 18–September 18)	Sex					
		Male		−0.034 (−0.081 to 0.012)	0.2	σ^2^	0.0002
		Female		0		τ_00_ _Individual_	0.001
		Temperature (°C)		−0.002 (−0.006 to 0.002)	0.42		
		Wind Speed (m/s)		−0.024 (−0.050 to 0.002)	0.07		
		Rainfall (mm^0.5^)		0.004 (−0.006 to 0.013)	0.44		
		Temp × Wind		0.0015 (0.0 to 0.003)	0.06		

CI, confidence interval.

## Discussion

Behavioral compensation via the regulation of activity patterns is an important mechanism by which animals balance energetic requirements across the day and year. Our results suggest that (1) the time of activity onset relative to photoperiod of male skunks, but not females, is relatively constant across seasonal stages, and other characteristics of activity timing (offset and period) in skunks may be more relevant to reproductive effort, (2) sex differences in reproductive requirements (e.g., conspecific competition for mates among males, lactation and care of young by females) lead to differences between the sexes in seasonal patterns of activity, and (3) environmental conditions, specifically ambient temperature and snow cover, influences total skunk activity only during the winter season.

### Activity timing

The timing of activity (chronotype) is likely a sexually selected trait in mammals (Hau et al. 2017), driven by competition among males to maximize reproductive fitness in the race to secure mates. This pattern has been described for European ground squirrels (*Spermophilus citellus*); males commence daily activity earlier than females during mating, presumably to secure access to reproductive females ahead of their later-rising counterparts (Everts et al. 2004). In contrast, we found that male skunks did not significantly change the timing of their daily activity onset relative to civil dusk across the year, and the timing of daily activity onset in males also did not significantly differ from that in females during the mating season. However, the cessation of activity was significantly later and the duration of daily activity bouts longer in males than females during mating. We suggest three possible explanations for the lack of earlier activity onsets during the mating season in male skunks when compared to the rest of the year. First, the significant increases in the overall duration and intensity of male activity bouts we documented during early spring compared to activity in winter and early fall may be sufficient for monitoring the locations of and securing mates, and the additional energetic costs and potential predation risks associated with early activity may outweigh the potential benefits. Second, other signals or characteristics of male quality related to pre- or post copulatory mate choice (e.g., body size or sperm competition) may be more important drivers of mate selection than male availability (earlier onset) alone. Third, the investment of locomotor activity toward the later part of the night, rather than the beginning, may be more advantageous for male skunks. We found that the males terminated activity significantly later than females during mating and that the intensity of male activity during mating peaks before dawn, suggesting that more dynamic social contexts may be influencing locomotor activity in striped skunks. It is possible that brief mate-guarding behavior toward the end of the night plays a more important role in mating success than earlier activity onset. Alternatively, because it is not uncommon for male and female skunks to share diurnal dens (Theimer et al. 2016), it may have been advantageous for males to increase activity toward the end of the night to secure a shared diurnal den with females that could increase opportunity for mating during the day or the following night.

Onset and cessation of activity in female skunks varied relative to civil twilight across seasons. Activity onset of female skunks varied seasonally, with activity beginning after dusk in the winter and spring, and before dusk in the summer and fall. In the summer, during lactation, females also terminated activity later than other times of year, suggesting that the energetic demands of lactation may drive activity timing in female skunks. In contrast, female skunks studied farther north (in Sakatchewan, Canada, 52° N), showed no consistent difference in activity onset or cessation times between spring (April–May) and late summer (August) (Larivière and Messier 1997), even though there are fewer hours of darkness available for activity at that higher latitude. Seasonal plasticity in nightly onsets of activity have also been demonstrated in Ord’s kangaroo rats (*Dipodomys ordii*) and are likely a consequence of seasonal trade-offs between the costs of predation and the benefits of reproduction and food abundance (White and Geluso 2007). We hypothesize that the differences in activity onsets across seasonal stages observed in females is a result of thermoregulatory and reproductive drivers that vary in importance across the year. Female skunks likely benefit from energy savings during the colder months through decreased overall activity and expression of daily torpor (Hwang et al. 2007a; Theimer et al. 2017b) while males may remain more active in winter in order to monitor locations of female dens for future mating opportunities (Theimer et al. 2016). Despite the earlier activity onset and later activity offset (relative to civil twilight) of females during lactation, the duration of female activity bouts did not vary significantly across stages. Extension of female activity onset and offset into daytime hours in summer through fall is likely due to a need to compensate for the shorter duration of darkness in order to meet the energetic costs of rearing young. Females may be responding to reproductive demands by increasing the intensity, altering the timing, and reducing the fragmentation of their daily activity rather than through increases in the duration of their daily activity bouts. Lastly, while we suggest the observed sex-differences in daily timing are likely to be adaptive, it is possible these differences are simple byproducts of intrinsic changes in circulating sex hormones (reviewed in Lightfoot 2008) or sex-specific morphology of the master circadian clock in the superchiasmatic nucleus (reviewed in Yan and Silver 2016) with no apparent selective advantage.

### Seasonal activity patterns

In contrast to previous studies of striped skunks (Larivière and Messier 1997; Weissinger et al. 2009), we identified sex-specific differences in activity across seasons. This was likely due to continuous and extended data collected by the accelerometers, which provided a more thorough picture of activity in comparison to previous work, which utilized radio telemetry. We found that both male and female striped skunks remained active throughout the year, with total activity (ODBA) lowest during winter months for females. Male activity in winter was lower than in early spring, but no different than that in early fall. Reduced activity in winter is consistent with many studies of skunks in northern part of North America that documented extensive periods in winter dens (Allen and Shapton 1942; Verts 1967; Houseknecht and Tester 1978) and reduced movements (Sunquist 1974; Weissinger et al. 2009). Striped skunks living in colder climates may use daily bouts of shallow torpor during the winter months for energetic savings (Mutch and Aleksiuk 1977; Hwang et al. 2007a; Theimer et al. 2017b). Females in our study area are more likely than males to den communally and experience torpor (Theimer et al. 2016, 2017b), and this difference in denning and thermoregulatory behavior could explain the differences between the sexes in activity during the winter period. Males may also visit female dens during winter, perhaps to monitor locations of potential mates (Theimer et al. 2016), and this also could have contributed to total higher activity in males during winter.

During early spring, all males we captured had descended testes, consistent with mating occurring at that time, as is typical for striped skunks (Verts 1967, Wade-Smith and Verts 1982). High ODBA in males during this period was therefore likely associated with increased energetic requirements from mate-seeking forays, intrasexual competition, and foraging. Female ODBA in early spring was similar to that in early fall, but was highest during the energetically demanding stage of lactation/young at heel. Although we were unable to gather data on male activity when females were in lactation, studies of skunk activity elsewhere found that activity of male skunks did not vary from April through August (Larivière and Messier 1997). Along with earlier activity onsets and increases in the magnitude of activity, the high activity levels of female skunks during lactation may also be attributed to changes in their daily activity rhythms. During lactation, females shifted from the highly variable nightly activity rhythms typical of both sexes through much of the year to prolonged and continuous bouts of nocturnal activity. In addition to reflecting increased foraging activity to meet increased energetic demands, this increased and sustained activity may be a consequence of female skunks being tied to a single maternal denning location during the rearing period. Whereas during other times of the year skunks may move more broadly and den opportunistically (Houseknecht and Tester 1978; Hwang et al. 2007b; Theimer et al. 2016), after parturition and throughout lactation, female skunks exhibit strong den fidelity, typically using a single den site until young disperse (Larivière and Messier 1997; 1998). To accommodate this change in denning behavior, female skunks may have utilized a central place foraging strategy during lactation, making repetitive forays across distances throughout the night to take advantage of resource-rich foraging locations, for example, bird feeders (Theimer et al. 2017a). However, it is important to note that activity estimated through ODBA cannot discern the difference between displacement and distance covered, and total female ODBA may still remain higher if the scalar quantity of distance traveled was greater than in males. It would be of interest for future studies to associate ODBA with fine-scale behavioral states (e.g., walking vs. running) to better distinguish the context of low versus high energy behaviors across the day.

### Environmental effects

Because environmental conditions greatly impact the thermoregulatory costs and total energy expenditure of mammals, there is widespread evidence that weather significantly affects the activity patterns of free living animals (Getz 1961; Long et al 2005; Williams et al. 2016b). For example, over 50% of the variation in daily activity of arctic ground squirrels (*Urocitellus parryii*) can be explained by daily variation in weather conditions (Williams et al. 2016b). Here, we show that weather conditions affected skunk activity differentially across the seasons. Weather was associated with daily ODBA only during the winter, with low temperatures and snow-cover negatively affecting daily ODBA. Ambient air temperatures were below the lower critical temperature of skunks (17°C) (Knudsen 1979) only during the winter period, and thermoregulatory conditions along with reduced foraging opportunities and increased cost of locomotion from snow cover likely combine to act as the major driver of daily activity. Several authors have proposed that deep snow rather than ambient temperature may be the major factor limiting movements of skunks in winter (Verts 1967; Sunquist 1974; Mutch and Aleksiuk 1977; Theimer et al. 2016). In red foxes (*Vulpes fulva*) in south-central Wisconsin, the combined effect of weather factors on fox activity was also greatest during winter, but precipitation was also the most important factor influencing activity during summer (Ables 1969). We found no effect of precipitation on total skunk activity in summer. However, we show that it may be possible that skunks are altering the timing of their daily activity (i.e., activity offset and period) to avoid unfavorable weather conditions during warmer months, rather than altering their total daily activity levels.

## Conclusions

Generally, thermoregulatory conditions and forage availability significantly constrain the time–energy budgets of species. As these factors vary seasonally, animals modulate their activity patterns to balance between environmental and biotic demands. Collectively, our results highlight the relative importance of behavioral thermoregulation and reproductive effort across both the daily and seasonal cycle in a primarily nocturnal mesocarnivore. Due to the challenges associated with traditional monitoring techniques, few studies have successfully examined the timing and fine-scale activity patterns of mammals in the field. Our results demonstrate the efficacy of accelerometers in revealing previously difficult-to-detect sex differences in a free-living mammal, and emphasizes the potential for sex-specific differences in chronobiology (Fendo and Mintz 2015).

## Author contributions

V.Y.Z., C.T.W., and C.L.B. contributed to the development of the concept. V.Y.Z., T.C.T., and C.T.W. captured animals and collected field data. T.C.T. provided logistical support and helped with permitting. V.Y.Z. analyzed data, drafted figures, and wrote the initial manuscript with input from C.T.W. and C.L.B. All authors contributed to revisions of the manuscript.

## Supplementary Material

obz013_Supplementary_Data

## References

[obz013-B1] Ables ED. 1969 Activity studies of red foxes in southern Wisconsin. J Wildl Manage 33:145–53.

[obz013-B2] Allen DL, Shapton WW. 1942 An ecological study of winter dens, with special reference to the eastern skunk. Ecology 23:59–68.

[obz013-B3] Brown DD, Kays R, Wikelski M, Wilson R, Klimley AP. 2013 Observing the unwatchable through acceleration logging of animal behavior. Anim Biotelem 1:20.

[obz013-B4] Cooper CF. 1960 Changes in vegetation, structure, and growth of southwestern pine forests since white settlement. Ecol Monogr 30:129–64.

[obz013-B5] Daan S, Aschoff J. 1982 Circadian contributions to survival In: Aschoff J, Daan S, Groos G, editors. Vertebrate circadian systems. Proceedings in Life Sciences. Berlin, Heidelberg: Springer.

[obz013-B6] Erlinge S, Sandell M. 1986 Seasonal changes in the social organization of male stoats, *Mustela erminea*: an effect of shifts between two decisive resources. Oikos 47:57–62.

[obz013-B7] Ernst CH. 1965 Rutting activities in a captive striped skunk. J Mammal 46:702–03.

[obz013-B8] Everts LG, Strijkstra AM, Hut RA, Hoffmann IE, Millesi E. 2004 Seasonal variation in daily activity patterns of free-ranging European ground squirrels (*Spermophilus citellus*). Chronobiol Int 21:57–71. 15129824 10.1081/cbi-120027982

[obz013-B9] Fendo JA, Mintz EM. 2015 Sex differences in behavioral circadian rhythms in laboratory rodents. Front Endocrinol 5:234. 10.3389/fendo.2014.00234 PMC4288375 25620955

[obz013-B10] Fletcher QE, Speakman JR, Boutin S, McAdam AG, Woods SB, Humphries MM. 2012 Seasonal stage differences overwhelm environmental and individual factors as determinants of energy expenditure in free‐ranging red squirrels. Funct Ecol 26:677–87.

[obz013-B11] Forrest J, Miller-Rushing AJ. 2010 Toward a synthetic understanding of the role of phenology in ecology and evolution. Philos Trans R Soc B 365:3101–12. 10.1098/rstb.2010.0145 PMC2981948 20819806

[obz013-B12] Geisser H, Reyer HU. 2005 The influence of food and temperature on population density of wild boar *Sus scrofa* in the Thurgau (Switzerland). J Zool 267:89–96.

[obz013-B13] Getz LL. 1961 Responses of small mammals to live-traps and weather conditions. Am Midl Nat 66:160–70.

[obz013-B14] Gittleman JL, Thompson SD. 1988 Energy allocation in mammalian reproduction. Am Zool 28: 863–75.

[obz013-B15] Halsey LG, Green JA, Wilson RP, Frappell PB. 2008 Accelerometry to estimate energy expenditure during activity: best practice with data loggers. Physiol Biochem Zool 82:396-404. 10.1086/589815 19018696

[obz013-B16] Halsey LG, Jones TT, Jones DR, Liebsch N, Booth DT. 2011 Measuring energy expenditure in sub-adult and hatchling sea turtles via accelerometry. PLoS One 6:e22311. 21829613 10.1371/journal.pone.0022311 PMC3150346

[obz013-B17] Halsey LG, Shepard ELC, Quintana F, Laich AG, Green JA, Wilson RP. 2009 The relationship between oxygen consumption and body acceleration in a range of species. Comp Biochem Physiol A Mol Integr Physiol 152:197–202. 18854225 10.1016/j.cbpa.2008.09.021

[obz013-B18] Halsey LG, White CR. 2010 Measuring energetics and behavior using accelerometry in cane toads Bufo marinus. PLoS One 5:e10170. 20422048 10.1371/journal.pone.0010170 PMC2858044

[obz013-B19] Hau M, Dominoni D, Casagrande S, Buck CL, Wagner G, Hazlerigg D, Greives T, Hut RA. 2017 Timing as a sexually selected trait: the right mate at the right moment. Philos Trans R Soc B 372:20160249. 10.1098/rstb.2016.0249 PMC5647276 28993493

[obz013-B20] Houseknecht CR, Tester JR. 1978 Denning habits of striped skunks (*Mephitis mephitis*). Am Midl Nat 100:424–30.

[obz013-B21] Horne JA, Östberg O. 1976 A self-assessment questionnaire to determine morningness-eveningness in human circadian rhythms. Int J Chronobiol 4:97–110. 1027738

[obz013-B22] Hut RA, Pilorz V, Boerema AS, Strijkstra AM, Daan S. 2011 Working for food shifts nocturnal mouse activity into the day. PLoS One 6:e17527. 21479166 10.1371/journal.pone.0017527 PMC3068156

[obz013-B23] Hwang YT, Larivière S, Messier F. 2007a Energetic consequences and ecological significance of heterothermy and social thermoregulation in striped skunks (*Mephitis mephitis*). Physiol Biochem Zool 80:138–45. 10.1086/509211 17160886

[obz013-B24] Hwang YT, Larivière S, Messier F. 2007b Local-and landscape-level den selection of striped skunks on the Canadian prairies. Can J Zool 85:33–9.

[obz013-B25] Kempenaers B, Borgström P, Loës P, Schlicht E, Valcu M. 2010 Artificial night lighting affects dawn song, extra-pair siring success, and lay date in songbirds. Curr Biol 20:1735–9. 20850324 10.1016/j.cub.2010.08.028

[obz013-B26] Kenagy GJ, Sharbaugh SM, Nagy KA. 1989 Annual cycle of energy and time expenditure in a golden-mantled ground squirrel population. Oecologia 78:269–82. 28312369 10.1007/BF00377166

[obz013-B27] Kenagy G. 2002 A time-energy analysis of daytime surface activity in degus, *Octodon degus**.* Rev Chil Hist Nat 75:149–56.

[obz013-B28] Knudsen KL. 1979 Temperature regulation in skunks (*Mephitis mephitis* and *Spilogale putorius*): Re-examination of metabolism and body size in mustelids [MA thesis]: University of Montana, Missoula.

[obz013-B29] Krebs JR, Kacelnik A. 1983 The dawn chorus in the great tit (*Parus major*): proximate and ultimate causes. Behaviour 83: 287–308.

[obz013-B30] Kuznetsova A, Brockhoff PB, Christensen RHB. 2017 lmerTest Package: Tests in Linear Mixed Effects Models (lmer objects of lme4 package). (http://CRAN.R-project.org/package=lmerTest)

[obz013-B31] Larivière S, Messier F. 1997 Seasonal and daily activity patterns of striped skunks (*Mephitis mephitis*) in the Canadian prairies. J Zool 243:255–62.

[obz013-B32] Larivière S, Messier F. 1998 Denning ecology of the striped skunk in the Canadian prairies: implications for waterfowl nest predation. J Appl Ecol 35:207–13.

[obz013-B33] Lee TN, Fridinger RW, Barnes BM, Buck CL, O'Brien DM. 2011 Estimating lean mass over a wide range of body composition: a calibration of deuterium dilution in the arctic ground squirrel. Rapid Commun Mass Spectrom 25:3491–6. 22095496 10.1002/rcm.5253

[obz013-B34] Lightfoot JT. 2008 Sex hormones’ regulation of rodent physical activity: a review. Int J Biol Sci 4:126. 18449357 10.7150/ijbs.4.126 PMC2359866

[obz013-B35] Logan M, Sanson GD. 2003 The effects of lactation on the feeding behaviour and activity patterns of free-ranging female koalas (*Phascolarctos cinereus* Goldfuss). Aust J Zool 51:415–28.

[obz013-B36] Long RA, Martin TJ, Barnes BM. 2005 Body temperature and activity patterns in free-living arctic ground squirrels. J Mammal 86:314–22.

[obz013-B37] Massei G, Genov PV, Staines BW. 1996 Diet, food availability and reproduction of wild boar in a Mediterranean coastal area. Acta Theriol 41:307–20.

[obz013-B38] Mutch GR, Aleksiuk M. 1977 Ecological aspects of winter dormancy in the striped skunk (*Mephitis mephitis*). Can J Zool 55:607–15.

[obz013-B39] Narasimhan R. 2017 Get Weather Data from the Web (http://ram-n.github.io/weatherData/)

[obz013-B40] Neiswenter SA, Dowler RC, Young JH. 2010 Activity patterns of two sympatric species of skunks (*Mephitis mephitis* and *Spilogale gracilis*) in Texas. Southwest Nat 55:16–21.

[obz013-B41] O’Farrell MJ. 1974 Seasonal activity patterns of rodents in a sagebrush community. J Mammal 55:809–23.

[obz013-B42] Owen-Smith N. 1998 How high ambient temperature affects the daily activity and foraging time of a subtropical ungulate, the greater kudu (*Tragelaphus strepsiceros*). J Zool 246:183–92.

[obz013-B43] Pagano AM, Williams TM. 2019 Estimating the energy expenditure of free-ranging polar bears using tri-axial accelerometers: a validation with doubly labeled water. Ecol Evol. 9:4210–9. 10.1002/ece3.5053 PMC6468055 31015999

[obz013-B44] Persson J. 2005 Female wolverine (*Gulo gulo*) reproduction: reproductive costs and winter food availability. Can J Zool 83:1453–9.

[obz013-B45] Poesel A, Kunc HP, Foerster K, Johnsen A, Kempenaers B. 2006 Early birds are sexy: male age, dawn song and extrapair paternity in blue tits, *Cyanistes* (formerly Parus) *caeruleus*. Anim Behav 72:531–8.

[obz013-B46] Raine RM. 1983 Winter habitat use and responses to snow cover of fisher (*Martes pennanti*) and marten (*Martes americana*) in southeastern Manitoba. Can J Zool 61:25–34.

[obz013-B47] Roenneberg T, Kuehnle T, Juda M., Kantermann T, Allebrandt K, Gordijn M, Merrow M. 2007 Epidemiology of the human circadian clock. Sleep Med Rev 11:429–38. 17936039 10.1016/j.smrv.2007.07.005

[obz013-B48] Ruf T, Geiser F. 2015 Daily torpor and hibernation in birds and mammals. Biol Rev 90:891–926. 25123049 10.1111/brv.12137 PMC4351926

[obz013-B49] Schmid B, Helfrich-Förster C, Yoshii T. 2011 A new ImageJ plug-in “ActogramJ” for chronobiological analyses. J Biol Rhythms 26:464–7. 21921300 10.1177/0748730411414264

[obz013-B50] Sikes RS, Animal Care and Use Committee of the American Society of Mammalogists. 2016 2016 Guidelines of the American Society of Mammalogists for the use of wild mammals in research and education. J Mammal 97:663–88. 29692469 10.1093/jmammal/gyw078 PMC5909806

[obz013-B51] Staudenmaier M Jr, Preston R, Sorenson P, Johndrow J. 2014. Climate of Flagstsaff, Arizona (Revision 7). NOAA Technical Memorandum NWS WR-273.

[obz013-B52] Storm GL. 1972 Daytime retreats and movements of skunks on farmlands in Illinois. J Wildl Manage 36:31–45.

[obz013-B53] Sunquist ME. 1974 Winter activity of striped skunks (*Mephitis mephitis*) in east-central Minnesota. Am Midl Nat 92:434–46.

[obz013-B54] Tachinardi P, Valentinuzzi VS, Oda GA, Buck CL. 2017 The interplay of energy balance and daily timing of activity in a subterranean rodent: a laboratory and field approach. Physiol Biochem Zool 90: 546–52. 28665185 10.1086/693003

[obz013-B55] Terrien J, Perret M, Aujard F. 2011 Behavioral thermoregulation in mammals: a review. Front Biosci 16:1428–44. 10.2741/3797 21196240

[obz013-B56] Theimer TC, Maestas JM, Bergman DL. 2016 Social contacts and den sharing among suburban striped skunks during summer, autumn, and winter. J Mammal 97:1272–81.

[obz013-B57] Theimer TC, Talk T, Johnson S, Bergman DL. 2017a Bird feeders as locations for skunk uptake of oral rabies vaccine baits. J Wildl Dis 53:424–7. 28151083 10.7589/2016-11-245

[obz013-B58] Theimer TC, Williams CT, Johnson SR, Gilbert AT, Bergman DL, Buck CL. 2017b Den use and heterothermy during winter in free-living, suburban striped skunks. J Mammal 98:867–73.

[obz013-B59] van der Vinne V, Gorter JA, Riede SJ, Hut RA. 2015 Diurnality as an energy-saving strategy: energetic consequences of temporal niche switching in small mammals. J Exp Biol 218:2585–93. 26290592 10.1242/jeb.119354

[obz013-B60] Verts BJ. 1967 The biology of the striped skunk. Illinois: University of Illinois Press.

[obz013-B61] Wade-Smith J, Verts BJ. 1982 Mephitis mephitis. Mamm Species 173:1–7.

[obz013-B62] Weissinger MD, Theimer TC, Bergman DL, Deliberto TJ. 2009 Nightly and seasonal movements, seasonal home range, and focal location photo-monitoring of urban striped skunks (*Mephitis mephitis*): implications for rabies transmission. J Wildl Dis 45:388–97. 19395748 10.7589/0090-3558-45.2.388

[obz013-B63] White JA, Geluso K. 2007 Seasonal differences in onset of surface activity of Ord's kangaroo rat (*Dipodomys ordii*). J Mammal 88:234–40.

[obz013-B64] Wilson RP, White CR, Quintana F, Halsey LG, Liebsch N, Martin GR, Butler PJ. 2006 Moving towards acceleration for estimates of activity-specific metabolic rate in free-living animals: the case of the cormorant. J Anim Ecol 75:1081–90. 16922843 10.1111/j.1365-2656.2006.01127.x

[obz013-B65] Williams CT, Wilsterman K, Kelley AD, Breton AR, Stark H, Humphries MM, Mcadam AG, Barnes BM, Boutin S, Buck CL. 2014 Light loggers reveal weather-driven changes in the daily activity patterns of arboreal and semifossorial rodents. J Mammal 95:1230–9.

[obz013-B66] Williams CT, Barnes BM, Buck CL. 2016a Integrating physiology, behavior, and energetics: biologging in a free-living arctic hibernator. Comp Biochem Physiol A Mol Integr Physiol 202:53–62. 27139082 10.1016/j.cbpa.2016.04.020

[obz013-B67] Williams CT, Wilsterman K, Zhang V, Moore J, Barnes BM, Buck CL. 2016b The secret life of ground squirrels: accelerometry reveals sex-dependent plasticity in above-ground activity. R Soc Open Sci 3:160404. 27703706 10.1098/rsos.160404 PMC5043325

[obz013-B68] Yan L, Silver R. 2016 Neuroendocrine underpinnings of sex differences in circadian timing systems. J Steroid Biochem Mol Biol 160:118–26. 26472554 10.1016/j.jsbmb.2015.10.007 PMC4841755

