# Supplementary material for: Reproductive and Environmental Drivers of Time and Activity Budgets of Striped Skunks
Source: Integr Org Biol. 2019 Jun 14;1(1):obz013. doi: 10.1093/iob/obz013 (PMC7671141; doi:10.1093/iob/obz013)
Supplement: obz013_Supplementary_Data [file obz013_supplementary_data.zip › SUPPLEMENTARY TABLE S1.docx]

| Stage | Parameter | | Estimate[95% CI] | P-value | Random Effects | |
| --- | --- | --- | --- | --- | --- | --- |
| **(a)**  Winter/Torpor  (Nov 13 – Dec 30) | Sex | Male | -64.33[-110,-18.7] | 0.03 | σ^2^ | 8013 |
|  |  | Female | 0 |  | τ_00_ _Day of Year_ | 765 |
|  | Temperature (°C) | | 1.38[-9.3,12.1] | 0.80 | τ_00_ _Individual_ | 475 |
|  | Wind Speed (m/s) | | -6.4[-24.7,11.9] | 0.50 |  |  |
|  | Temp x Wind | | 0.96[-1.9,3.8] | 0.51 |  |  |
| **(b)**  Mating  (Feb 29 – Mar 22) | Sex | Male | -26.4[-56,3.1] | 0.08 | σ^2^ | 4662 |
|  |  | Female | 0 |  |  |  |
|  | Temperature (°C) | | -1.9[-12.1,8.23] | 0.71 |  |  |
|  | Wind Speed (m/s) | | -10[-39.8,19.8] | 0.51 |  |  |
|  | Rainfall (mm^0.5^) | | 22.1[-118,162] | 0.76 |  |  |
|  | Temp x Wind | | 0.45[-2.3,3.2] | 0.75 |  |  |
| **(c)**  Lactation/at Heel; Females only  (Jun 26 – Jul 26) | Temperature (°C) | | 2.48[-0.81,5.78] | 0.14 | σ^2^ | 567 |
|  | Wind Speed (m/s) | | -5.97[-34.2,22.3] | 0.68 | τ_00_ _Individual_ | 184 |
|  | Rainfall (mm^0.5^) | | 68.7[20.2,117.2] | 0.01 |  |  |
|  | Temp x Wind | | 0.20[-1.07,1.47] | 0.76 |  |  |
| **(d)**  Fattening/Dispersal  (Aug 18 – Sep 18) | Sex | Male | 22.5[6.5,38.4] | 0.01 | σ^2^ | 2096 |
|  |  | Female | 0 |  |  |  |
|  | Temperature (°C) | | 3.78 [-4.0,11.5] | 0.34 |  |  |
|  | Wind Speed (m/s) | | 3.02[-65.4,71.4] | 0.93 |  |  |
|  | Rainfall (mm^0.5^) | | -19.9[-61.1,21.3] | 0.35 |  |  |
|  | Temp x Wind | | -0.26[-4.13,3.61] | 0.90 |  |  |
