# Supplementary material for: Reproductive and Environmental Drivers of Time and Activity Budgets of Striped Skunks
Source: Integr Org Biol. 2019 Jun 14;1(1):obz013. doi: 10.1093/iob/obz013 (PMC7671141; doi:10.1093/iob/obz013)
Supplement: obz013_Supplementary_Data [file obz013_supplementary_data.zip › SUPPLEMENTARY TABLE S2.docx]

| Stage | Parameter | | Estimate[95% CI] | P-value | Random Effects | |
| --- | --- | --- | --- | --- | --- | --- |
| **(a)**  Winter/Torpor  (Nov 13 – Dec 30) | Sex | Male | 0.46[-0.49,1.4] | 0.36 | σ^2^ | 3.27 |
|  |  | Female | 0 |  | τ_00_ _Day of Year_ | 0.04 |
|  | Temperature (°C) | | -0.04[-0.24,0.17] | 0.74 | τ_00_ _Individual_ | 0.23 |
|  | Wind Speed (m/s) | | -0.21[-0.49,0.07] | 0.15 |  |  |
|  | Temp x Wind | | 0.03[-0.02,0.08] | 0.23 |  |  |
| **(b)**  Mating  (Feb 29 – Mar 22) | Sex | Male | 1.27[0.67,1.88] | <0.0001 | σ^2^ | 1.95 |
|  |  | Female | 0 |  |  |  |
|  | Temperature (°C) | | 0.11[-0.15,0.36] | 0.42 |  |  |
|  | Wind Speed (m/s) | | 0.27[-0.24,0.78] | 0.30 |  |  |
|  | Rainfall (mm^0.5^) | | -0.67[-4.76,3.42] | 0.75 |  |  |
|  | Temp x Wind | | -0.02[-0.08,0.04] | 0.49 |  |  |
| **(c)**  Lactation/at Heel; Females only  (Jun 26 – Jul 26) | Temperature (°C) | | -0.24[-0.40,-0.09] | 0.003 | σ^2^ | 0.39 |
|  | Wind Speed (m/s) | | -1.38[-2.35,-0.42] | 0.01 | τ_00_ _Individual_ | 0.041 |
|  | Rainfall (mm^0.5^) | | -0.71[-1.66,0.24] | 0.15 |  |  |
|  | Temp x Wind | | 0.06[0.02,0.11] | 0.01 |  |  |
| **(d)**  Fattening/Dispersal  (Aug 18 – Sep 18) | Sex | Male | -1.19[-2.02,-0.36] | 0.03 | σ^2^ | 0.71 |
|  |  | Female | 0 |  | τ_00_ _Day of Year_ | 0.001 |
|  | Temperature (°C) | | 0.13[-0.12,0.38] | 0.32 | τ_00_ _Individual_ | 0.21 |
|  | Wind Speed (m/s) | | 0.61[-0.98,2.20] | 0.46 |  |  |
|  | Rainfall (mm^0.5^) | | 0.28[-0.31,0.87] | 0.35 |  |  |
|  | Temp x Wind | | -0.03[-0.13,0.06] | 0.50 |  |  |
