# Supplementary material for: Reproductive and Environmental Drivers of Time and Activity Budgets of Striped Skunks
Source: Integr Org Biol. 2019 Jun 14;1(1):obz013. doi: 10.1093/iob/obz013 (PMC7671141; doi:10.1093/iob/obz013)
Supplement: obz013_Supplementary_Data [file obz013_supplementary_data.zip › SUPPLEMENTARY TABLE S3.docx]

| Stage | Parameter | | Estimate[95% CI] | P-value | Random Effects | |
| --- | --- | --- | --- | --- | --- | --- |
| **(a)**  Winter/Torpor  (Nov 13 – Dec 30) | Sex | Male | -46.8[-121,27.0] | 0.25 | σ^2^ | 6732 |
|  |  | Female | 0 |  | τ_00_ _Individual_ | 2299 |
|  | Temperature (°C) | | 0.4[-4.92,5.72] | 0.88 |  |  |
|  | Wind Speed (m/s) | | -5.4[-14.1,3.29] | 0.23 |  |  |
|  | Temp x Wind | | 2.51[0.60,4.41] | 0.01 |  |  |
| **(b)**  Mating  (Feb 29 – Mar 22) | Sex | Male | 44.3[14.3,74.2] | 0.001 | σ^2^ | 4913 |
|  |  | Female | 0 |  |  |  |
|  | Temperature (°C) | | 1.85[-5.84,9.54] | 0.64 |  |  |
|  | Wind Speed (m/s) | | -0.35[-13.6,12.9] | 0.96 |  |  |
|  | Temp x Wind | | 0.27[-2.13,2.66] | 0.83 |  |  |
| **(c)**  Lactation/at Heel; Females only  (Jun 26 – Jul 26) | Temperature (°C) | | -0.95[-10.2,8.27] | 0.84 | σ^2^ | 1039 |
|  | Wind Speed (m/s) | | -9.96[-72.3,52.4] | 0.76 | τ_00_ _Individual_ | 699 |
|  | Rainfall (mm^0.5^) | | -0.72[-41.4,40.0] | 0.97 |  |  |
|  | Temp x Wind | | 0.71[-3.28,4.71] | 0.73 |  |  |
| **(d)**  Fattening/Dispersal  (Aug 18 – Sep 18) | Sex | Male | -46[-86.5,-5.47] | 0.07 | σ^2^ | 1371 |
|  |  | Female | 0 |  | τ_00_ _Individual_ | 509 |
|  | Temperature (°C) | | 7.7 [4.41,11.0] | <0.0001 |  |  |
|  | Wind Speed (m/s) | | 34.1[4.21,64.0] | 0.03 |  |  |
|  | Rainfall (mm^0.5^) | | -29.7[-57.2,-2.18] | 0.04 |  |  |
|  | Temp x Wind | | -3.4[-5.88,-0.96] | 0.007 |  |  |
