# Supplementary figures and images for: Reproductive and Environmental Drivers of Time and Activity Budgets of Striped Skunks
Source: Integr Org Biol. 2019 Jun 14;1(1):obz013. doi: 10.1093/iob/obz013 (PMC7671141; doi:10.1093/iob/obz013)

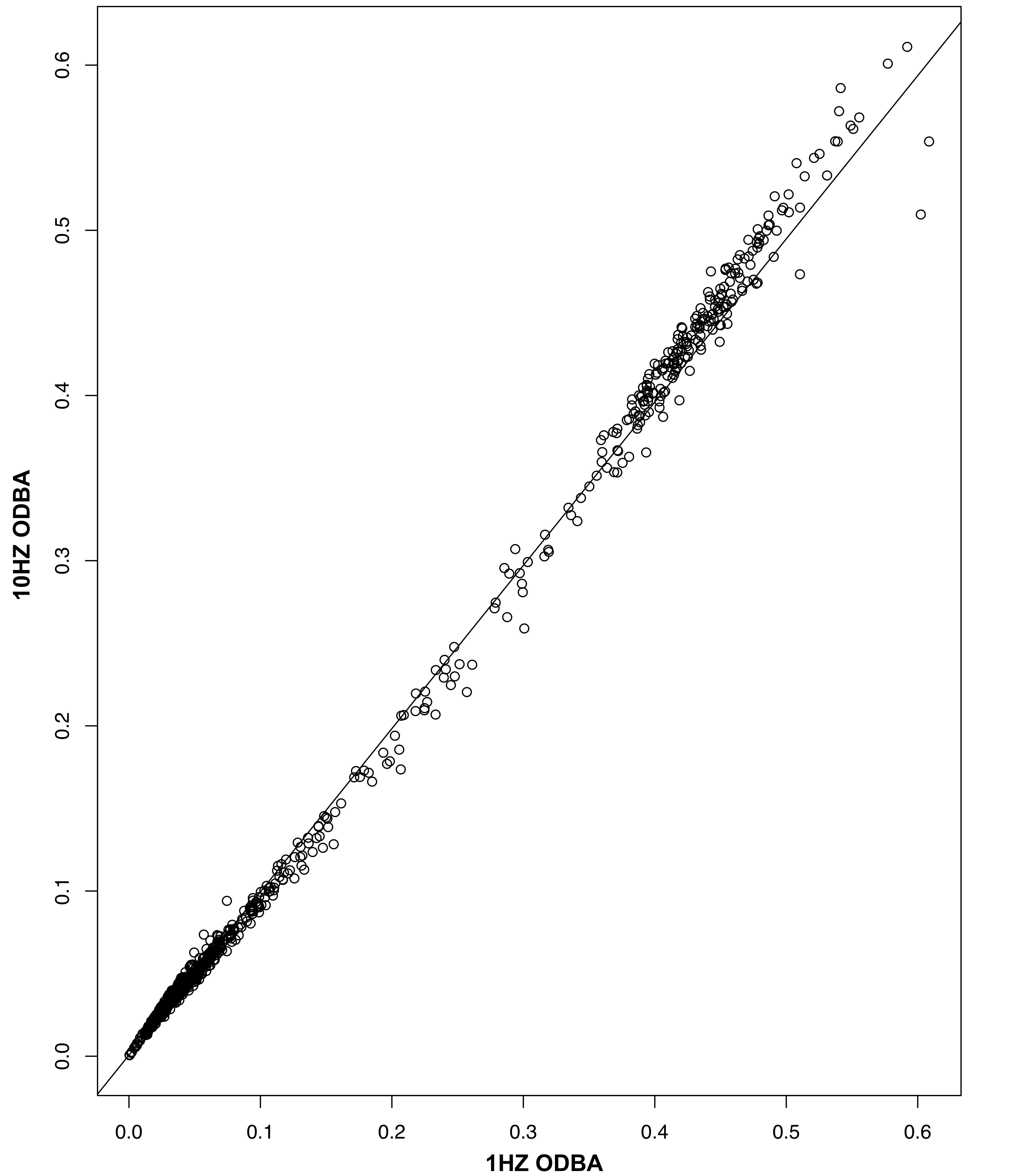

Supplement: obz013_Supplementary_Data [file obz013_supplementary_data.zip › SUPPLEMENTARY FIGURE S1.jpg]

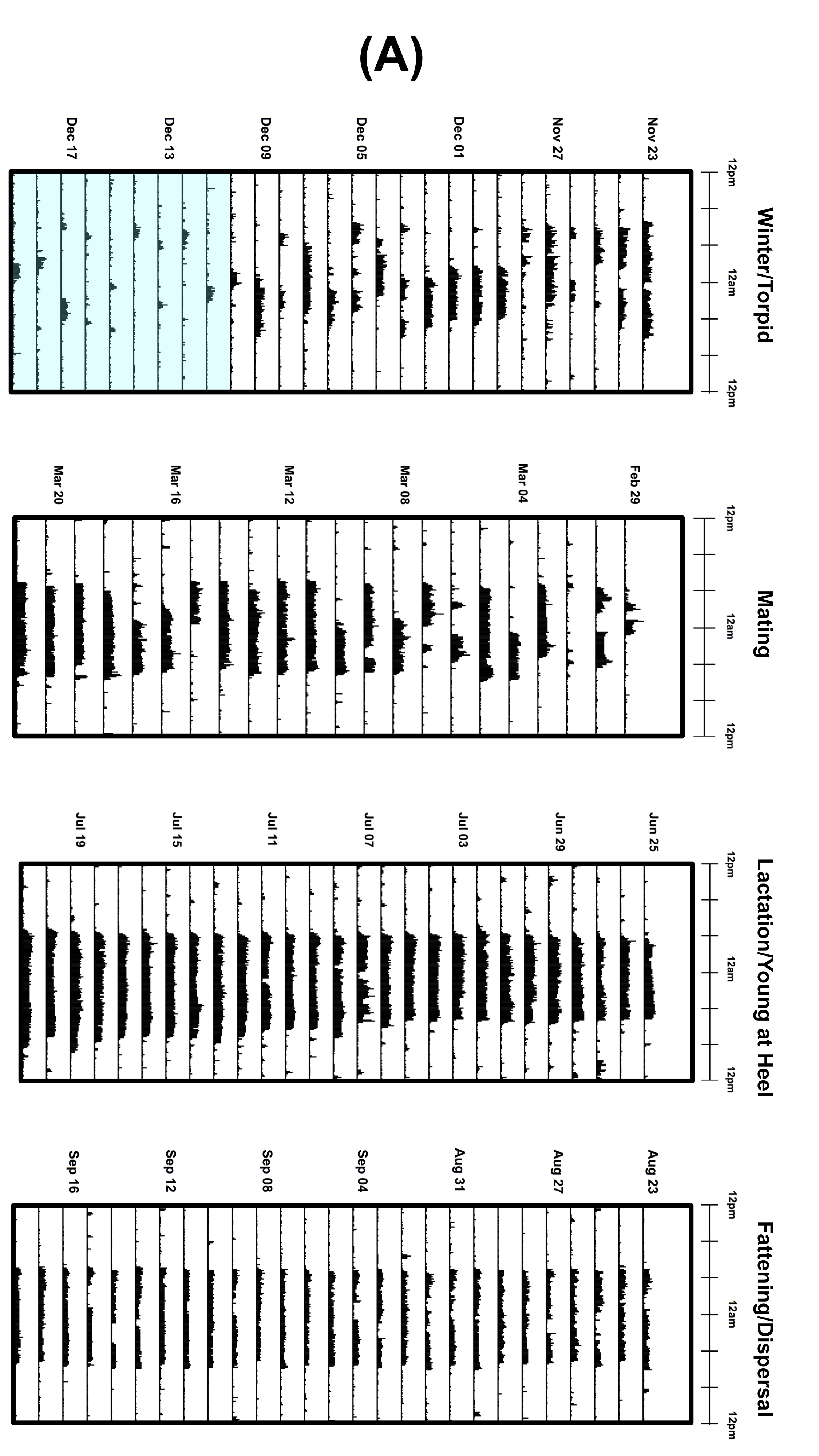

Supplement: obz013_Supplementary_Data [file obz013_supplementary_data.zip › SUPPLEMENTARY FIGURE S2A.jpg]

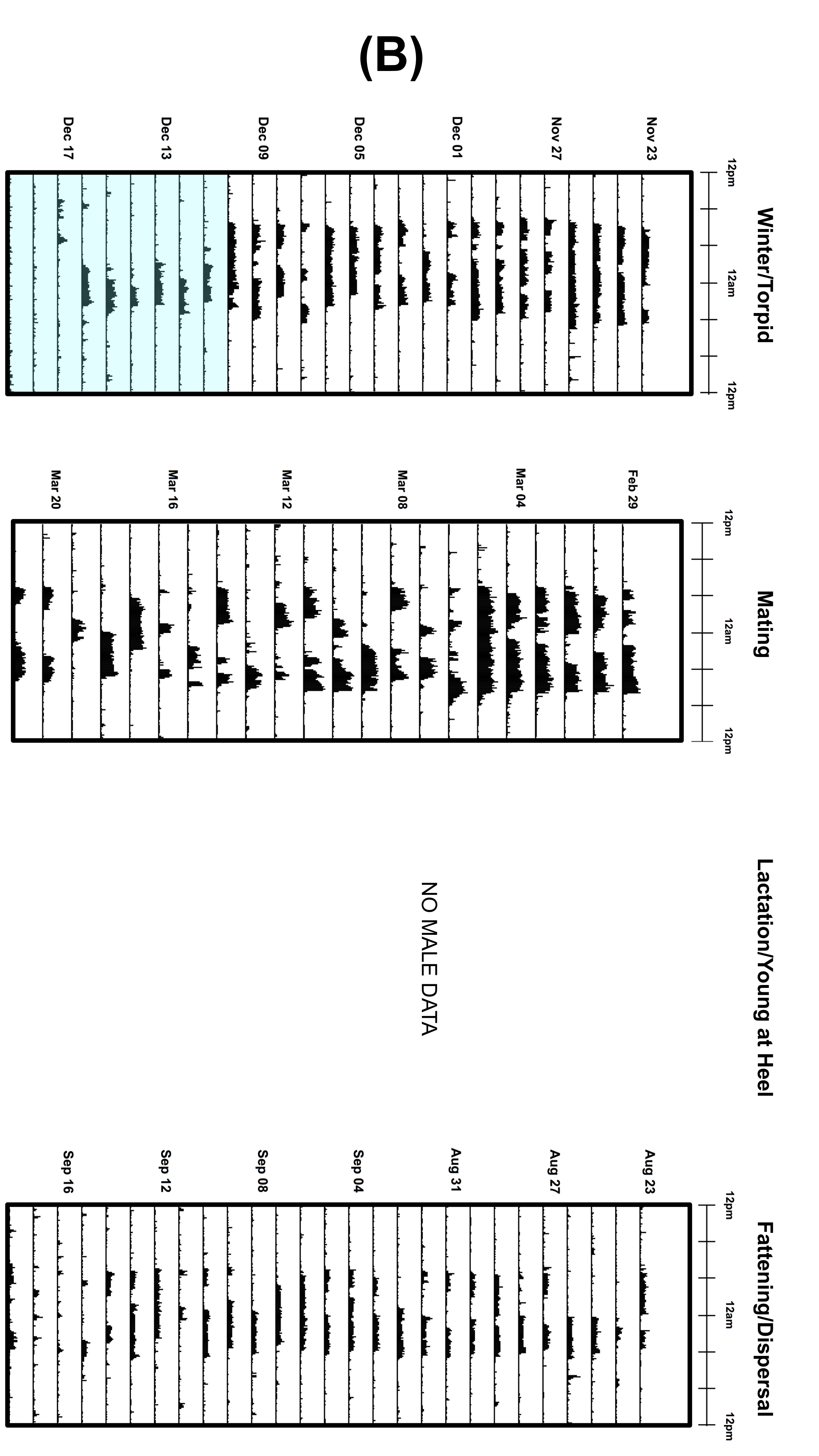

Supplement: obz013_Supplementary_Data [file obz013_supplementary_data.zip › SUPPLEMENTARY FIGURE S2B.jpg]

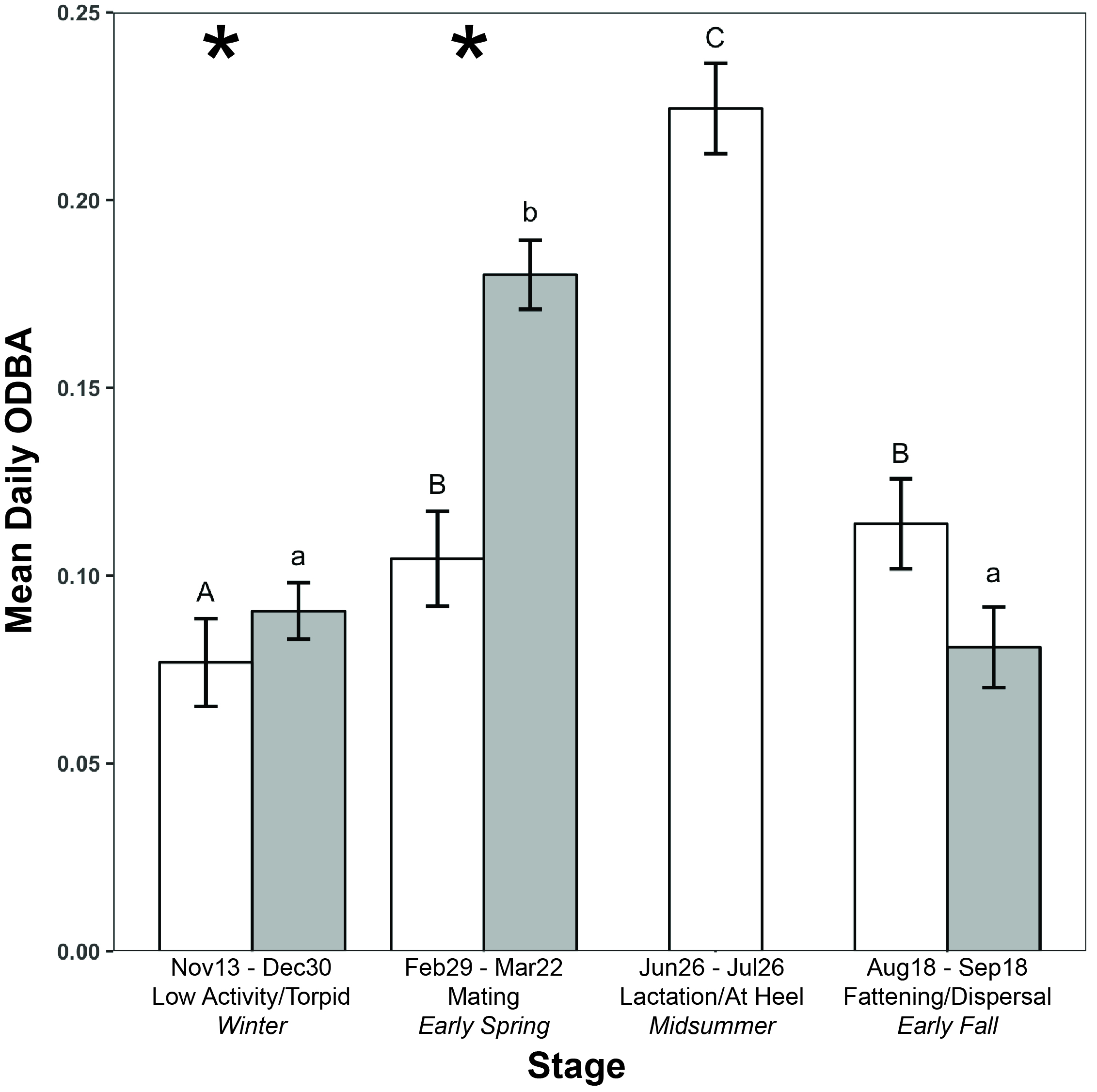

Supplement: obz013_Supplementary_Data [file obz013_supplementary_data.zip › SUPPLEMENTARY FIGURE S3.jpg]
